# Development and Validation of a Novel Prognostic Tool to Predict Recurrence of Paroxysmal Atrial Fibrillation after the First-Time Catheter Ablation: A Retrospective Cohort Study

**DOI:** 10.3390/diagnostics13061207

**Published:** 2023-03-22

**Authors:** Junjie Huang, Hao Chen, Quan Zhang, Rukai Yang, Shuai Peng, Zhijian Wu, Na Liu, Liang Tang, Zhenjiang Liu, Shenghua Zhou

**Affiliations:** Department of Cardiology, The Second Xiangya Hospital of Central South University, Changsha 410011, China

**Keywords:** paroxysmal atrial fibrillation, catheter ablation, recurrence, prediction model, prognostic tool, nomogram

## Abstract

There is no gold standard to tell frustrating outcomes after the catheter ablation of paroxysmal atrial fibrillation (PAF). The study aims to construct a prognostic tool. We retrospectively analyzed 315 patients with PAF who underwent first-time ablation at the Second Xiangya Hospital of Central South University. The endpoint was identified as any documented relapse of atrial tachyarrhythmia lasting longer than 30 s after the three-month blanking period. Univariate Cox regression analyzed eleven preablation parameters, followed by two supervised machine learning algorithms and stepwise regression to construct a nomogram internally validated. Five factors related to ablation failure were as follows: female sex, left atrial appendage emptying flow velocity ≤31 cm/s, estimated glomerular filtration rate <65.8 mL/(min·1.73 m^2^), P wave duration in lead aVF ≥ 120 ms, and that in lead V1 ≥ 100 ms, which constructed a nomogram. It was correlated with the CHA_2_DS_2_-VASc score but outperformed the latter evidently in discrimination and clinical utility, not to mention its robust performances in goodness-of-fit and calibration. In addition, the nomogram-based risk stratification could effectively separate ablation outcomes. Patients at risk of relapse after PAF ablation can be recognized at baseline using the proposed five-factor nomogram.

## 1. Introduction

Atrial fibrillation (AF) is a common arrhythmia posing a severe burden worldwide [1]. Catheter ablation of AF, particularly PAF, is becoming a pivotal therapeutic strategy. In addition, it has demonstrated superiority over medications in maintaining sinus rhythm and improving quality of life [2,3,4]. Notably, the relapse rate after successful ablation is still high [5,6,7], and patients at the stake of relapse cannot be accurately distinguished before ablation for lack of a gold standard.

Successful dissociation of the left atrium (LA) with pulmonary veins set the basis for PAF ablation. In addition, left atrial appendage (LAA) closure can offer additional prevention from systemic embolism. However, the enlargement and dysfunction of LA/LAA could strongly undermine the ablation efficacy [8,9,10]. Comorbidities such as hypertension, hyperthyroidism, and obesity, frequently seen in AF patients, have also been connected with LA enlargement and AF occurrences [11,12,13]. Modifiable lifestyles, such as smoking [14] and endurance exercises [15], additionally lift the AF incidence. Channelopathies, well-established to cause ventricular arrhythmias and cardiac arrests, also generate an arrhythmogenic substrate of atria, thus giving rise to AF [16,17]. Moreover, potential risk factors keep emerging, such as the female sex [18] and renal insufficiency [19,20]. Although several predictors have been established, any factor alone meets with limited predictive capacity or inconvenience to generalize. A robust prognostic tool compatible with a bedside setting is needed to promote predictive accuracy and guide reablation initiation.

Here, based on the evidence above, we report a five-factor nomogram predictive of AF relapse and its internal validation in 315 patients with PAF undergoing first-time catheter ablation.

## 2. Materials and Methods

### 2.1. Inclusion and Exclusion

**Figure 1:**
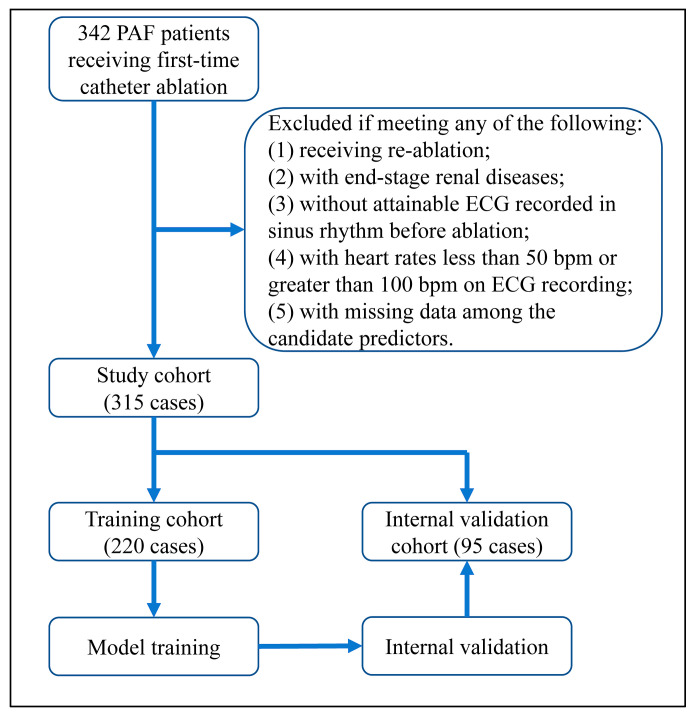
Flowchart of the research.

The research was a retrospective cohort study. Eleven variables were candidates for model construction, followed by the 10 EPV (10 events per variable) criterion to ensure the sample size. Obeying that, we initially included 342 patients who suffered non-valvular PAF and received cryoballoon or radiofrequency ablation from 2017 to 2022 at the Second Xiangya Hospital of Central South University. Patients were excluded if they had any of the following: (1) receiving reablation; (2) with end-stage renal diseases; (3) without attainable electrocardiogram (ECG) recorded in sinus rhythm before ablation; (4) with heart rates less than 50 bpm or greater than 100 bpm on ECG recording; (5) with missing data among the candidate predictors. Overall, 27 out of 342 cases met the exclusion criteria, as specified in Figure 1.

### 2.2. Ablation Procedure

PAF ablation was conducted after the exclusion of LAA thrombosis via trans-esophageal echocardiography. For isolating pulmonary veins from the LA substrate, the Carto^®^ or Ensite^TM^ three-dimensional mapping system navigated the catheter to ablate adjacent areas or the antrum. The bi-directional conduction block was seen as a success. The implementation of additional procedures, including superior vena cava isolation, linear ablation, and cavo-tricuspid isthmus ablation, were at the discretion of operators due to the corresponding findings. Patients were heparinized during operations, with the activated clotting time ranging from 250 to 350 s. If the endeavors above failed to terminate a spontaneous or induced AF state, synchronous direct current or medicative cardioversion would be accomplished selectively.

### 2.3. Observation

After ablation, patients were routinely followed up at 3, 6, 12 months, and thereafter, every six months by trained practitioners with limited knowledge of baseline data. Patients received ECG or Holter-ECG reviews every three months after ablation and at any suspected symptomatic episode. Anti-arrhythmia drugs (AAD) would be ceased three months after ablation if there was no evidence of relapse. Any documented episode of atrial tachyarrhythmia (ATa) relapse lasting longer than 30 s after the 3-month blanking period was seen as the endpoint. The shortest and longest observation durations were 182 (0.5 years) and 1831 days (5 years), respectively, with 1098 days (3 years) as the median.

### 2.4. Baseline Characteristics

Several variables were recorded as baseline characteristics: the CHA_2_DS_2_-VASc score, age at procedure, history of type 2 diabetes mellitus and stroke/transient ischemia attack (TIA), left ventricular end-diastolic volume (LVEDd), left ventricular ejection fraction (LVEF), level of N-terminal prohormone brain natriuretic peptide (NTproBNP), medications at baseline including angiotensin-converting enzyme inhibitors (ACEI)/angiotensin receptor blockers (ARB), beta-blockers, AADs, and novel oral anticoagulants (NOACs). For catheter ablation, we documented its energy type, procedural time, and additional procedures other than pulmonary vein isolations.

### 2.5. Candidate Predictors

Eleven variables were treated as candidates for model construction: sex, history of hypertension, body mass index (BMI), estimated glomerular filtration rate (eGFR), LA diameter, LAA emptying flow velocity, P wave duration (PWD) in the lead II, III, aVF, V1, and its terminal negative phase. All data were obtained preablation. The eGFR was calculated through the CKD-EPI equation [21]. LA diameter and LAA emptying flow velocity were measured by trans-thoracic or trans-esophageal echocardiography. On the standard recording, ECG was documented at least five half-lives after AAD discontinuation and then digitized. Independent practitioners, with limited awareness of baseline data and ablation outcomes, analyzed the echo- or electro-cardiographic parameters. MATLAB software (version R2022a) measured the P wave indices via 10-multiple magnification. 

### 2.6. Model Construction and Assessment

We set different strategies for the continuous variables. First, we used the R package *CatPredi* to find the optimal cut-points for the continuous variables using the *addfor* algorithm [22]. BMI, eGFR, LA diameter and LAA emptying flow velocity were then categorized. Second, to ease application, the P wave indices were categorized at the suboptimal cut-points, defined as those values closest to the optimal ones within the range of integral multiples of 20 ms (half of a little block). Categorical data were transferred through dummy coding.

For model construction, univariate Cox regression tested the candidates preliminarily. If they were highly correlated, the least absolute shrinkage and selection operator (lasso) [23] algorithm and a random survival forest (RSF) [24] would assist with variable selection. Stepwise regression was finally used, and the variables with a *p*-value less than 0.05 were selected to build a prediction model. A nomogram was then plotted to visualize it.

We assessed the nomogram’s performance among discrimination, goodness-of-fit, calibration, clinical utility, and separative efficacy. Time-dependent receiver operator characteristic (ROC) curves, the areas under the curves (AUC), and the c-index curves described its discriminative power. Akaike information criteria (AIC), Bayesian information criteria (BIC), and Brier-score curves illustrated its goodness-of-fit. Calibration plots depicted the prediction–observation deviations. The assessment above was corrected via bootstrap resampling. Decision curve analyses evaluated its clinical utility. Kaplan–Meier curves and a log-rank test assessed its risk stratification strategy. 

If there were competing risk scores for prediction, the integrated discrimination improvement index (IDI) and net reclassification index (NRI) would compare their discriminative performances via perturbation resampling. A weighted Kappa test evaluated the agreement between their risk stratification strategies. Mediation effect analysis illustrated the triangular relationship between the risk scores and time-to-events.

Model training and internal validation were accomplished by R (version 4.2.2).

### 2.7. Statistical Analyses

Descriptive statistics were performed among the relapse and censoring groups. Continuous variables were described with mean ± standard deviance or median (interquartile range). Categorical data were described with frequency (proportion). Student’s *t*-tests, Mann–Whitney U tests, and chi-square tests determined the groups’ comparability. Spearman’s correlation and a correlogram depicted the inter-predictor relationship. All *p*-values reported were two-sided, and those less than 0.05 were considered significant. All the statistical analyses were carried out by R (version 4.2.2).

## 3. Results

### 3.1. Baseline Characteristics and Categorization of Continuous Variables

With 315 patients finally included, 153 relapses occurred, thus the sample size meeting the criterion. We recapitulated baseline characteristics for the relapse and censoring groups (Table 1). 

Due to the missing values, NTproBNP was reported via categorization. Notably, nearly half of the patients (140, 44.4%) were recognized as lone AF. For most characteristics, the two groups remained comparable, whereas the relapse group had a higher CHA_2_DS_2_-VASc score, a higher proportion of female sex, slower LAA emptying flow velocity, and a wider PWD than the censoring group. 

We then randomly split the study population into the training and internal validation cohorts in a 7:3 ratio. For the continuous variables, the optimal cut-points were found as follows: BMI (26 kg/m^2^), LAA emptying flow velocity (31 cm/s), LA diameter (33 mm), eGFR (65.8 mL/(min·1.73 m^2^)), PWD in lead II (116.5 ms), III (116.3 ms), aVF (123.9 ms), V1 (103.4 ms), and its terminal negative phase (57.8 ms). Hence, the suboptimal cut-points for P wave indices were ascertained: 120 ms for PWD in the lead II, III, and aVF, 100 ms for PWD in lead V1, and 60 ms for its terminal negative phase.

### 3.2. Variable Selection and Model Construction

Univariate Cox regression selected eight risk factors: the female sex, LAA emptying flow velocity ≤31 cm/s, eGFR < 65.8 mL/(min·1.73 m^2^), PWD in lead II ≥ 120 ms, III ≥ 120 ms, aVF ≥ 120 ms, V1 ≥ 100 ms, and its terminal negative phase ≥60 ms, as specified in Table 2. 

**Figure 2:**
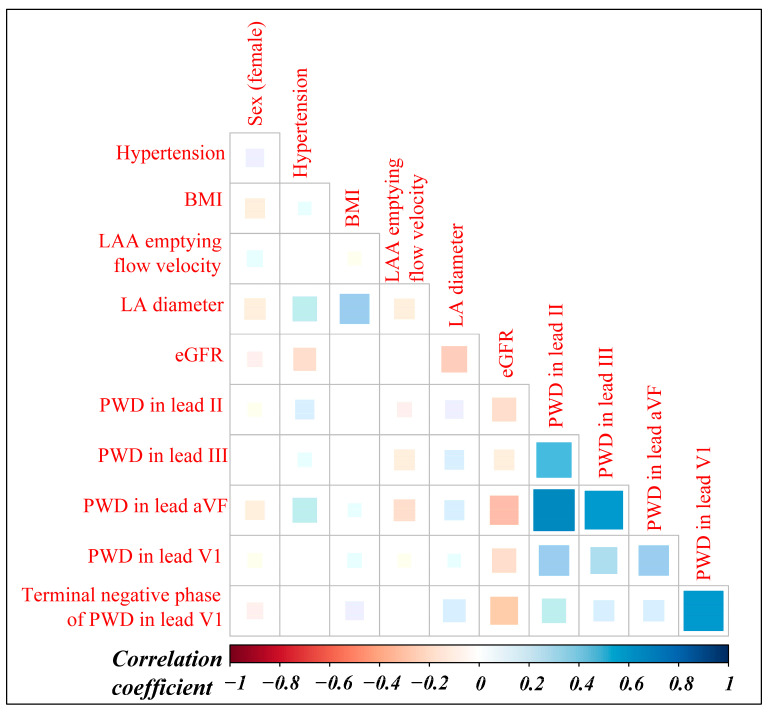
Correlogram of the candidate predictors.

As anticipated, the candidate predictors were strongly correlated, especially between P wave indices (Figure 2). 

**Figure 3:**
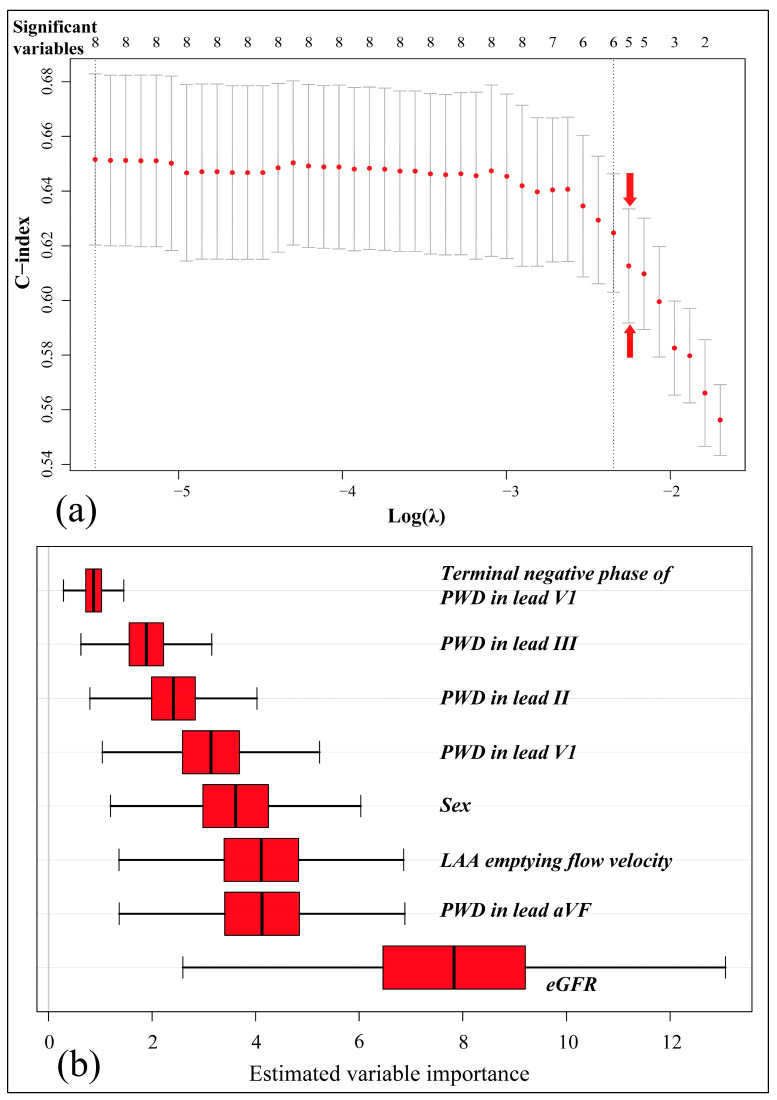
The Lasso-Cox model (**a**) and RSF (**b**) for variable selection. (**a**) depicts how the penalized c-index varied via 5-folded cross-validation, as the L1 norm (lambda value) increased. The logarithmic lambda value of −2.2535 (the red arrow) was chosen for variable selection. (**b**) gives the estimation of variable importance (with a 95% confidence interval) by RSF via a subsampled method.

Therefore, we used a Lasso-Cox model and an RSF for variable selection. A relatively large lambda value of 0.1050327 was chosen to constrain model complexity (Figure 3a), while the RSF estimated the variable importance via subsampled resampling (Figure 3b).

Under the L1 normalization, PWD in lead II, III, and the terminal negative phase of PWD in lead V1 were found incapable (Table 2), as reaffirmed by the RSF estimation (Figure 3b). Finally, a bi-directional stepwise regression allowed the remaining factors to construct a prediction model (Table 2).

### 3.3. The Five-Factor Nomogram and Its Application

**Figure 4:**
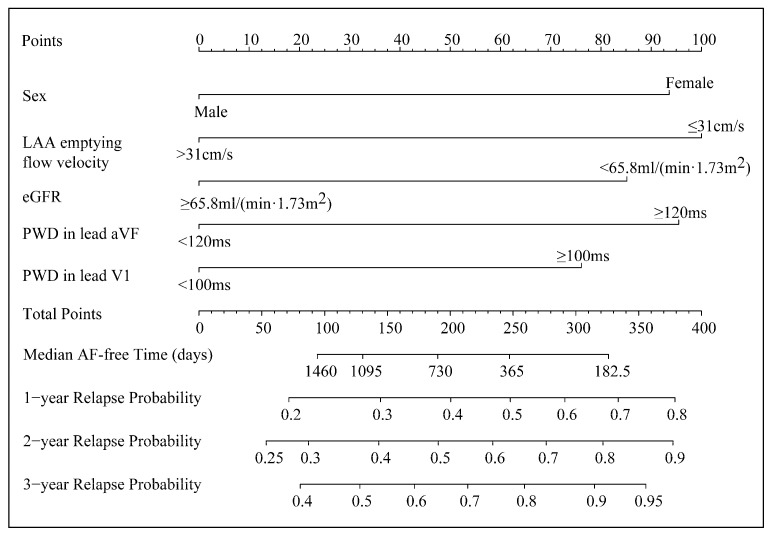
The five-factor nomogram.

A nomogram was plotted to represent the five-factor model (Figure 4).

Female sex, LAA emptying flow velocity ≤ 31 cm/s, eGFR < 65.8 mL/(min·1.73 m^2^), PWD in lead aVF ≥ 120 ms, and that in lead V1 ≥ 100 ms contributed 93.6, 100, 85.2, 95.5, and 76.1 points, respectively, to the total points of the nomogram. The total points corresponded to specific median relapse-free times and predicted probabilities of relapse across 1, 2, and 3 years after ablation. In addition, they served as evidence for risk stratification. It is worth noting that the transcendence of the predicted probabilities over the preset threshold probabilities led to a reablation in decision curve analyses, which also provided the reference for clinical decision-making.

### 3.4. Discriminative Power

**Figure 5:**
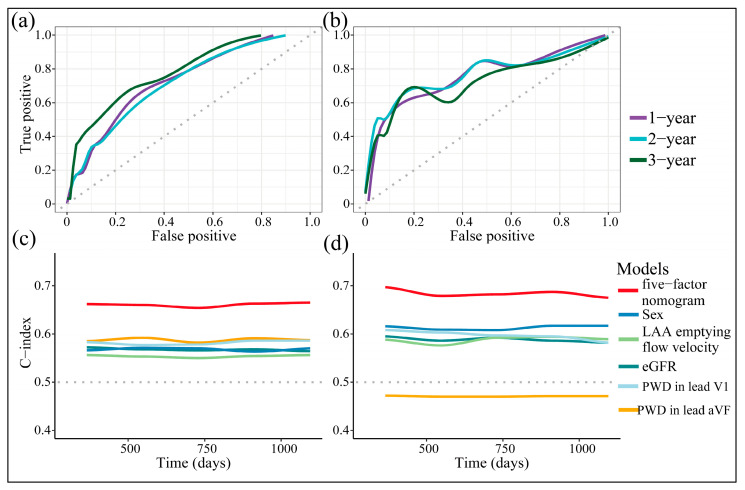
Time-dependent ROC curves across 1, 2, and 3 years after ablation (**a**) and internally validated (**b**), with c-index analyses (**c**) and internally validated (**d**).

We then assessed the discriminative power of the nomogram. It performed evenly at sensitivity and specificity across different thresholds of its total points (Figure 5a,b). 

The bootstrap-corrected AUC of the training cohort was 0.722, 0.711, 0.777 across 1, 2, and 3 years after ablation, respectively, and that internally validated was 0.755, 0.757, and 0.705. Similar results were yielded from c-index analyses (Figure 5c,d), demonstrating that the nomogram persistently outperformed any predictor alone in discriminative accuracy. The internal validation cohort was prone to having similar AUCs and c-indexes to the training one, setting the potential to generalize. In addition, the nomogram’s total points moderately correlated with the CHA_2_DS_2_-VASc score (correlation coefficient: 0.33, *p* < 0.0001), and the latter served as a competing risk score (hazard ratio (HR): 1.18, 95% confidence interval (CI) (1.08–1.30), *p* = 0.0006). However, the nomogram guaranteed remarkable improvement in discrimination, proving its superiority over the CHA_2_DS_2_-VASc score (Table 3).

### 3.5. The Goodness-of-Fit, Calibration, and Clinical Utility

We then evaluated the model’s goodness-of-fit. The AIC and BIC values were 1013.40 and 1030.37, respectively, which were lower than any predictor alone (Table 4). 

**Figure 6:**
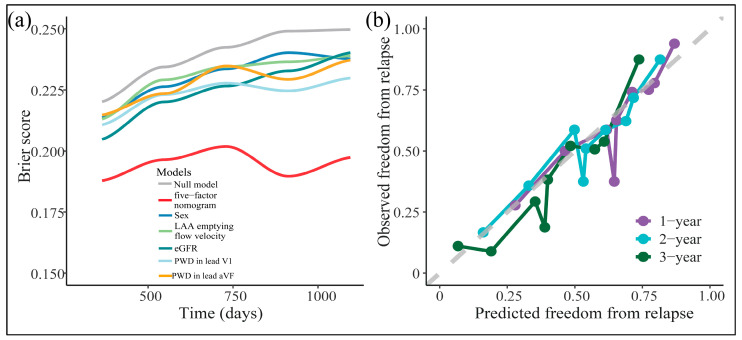
Time-dependent Brier score curves (**a**) and calibration plots (**b**) across 1, 2, and 3 years after ablation.

Parallelly, the nomogram had significantly lower Brier-scores than random guessing (null model) or any predictor alone (Figure 6a), rendering its goodness-of-fit. 

**Figure 7:**
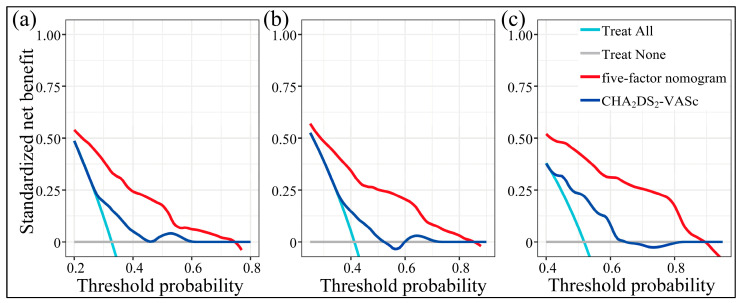
Decision curve analyses across 1 (**a**), 2 (**b**), and 3 years (**c**) after ablation.

Moreover, its prediction fitted nicely with the observation across the majority scale of predicted probabilities (Figure 6b). Across the reasonable threshold probabilities, the nomogram guaranteed higher net benefits than default decisions or the CHA_2_DS_2_-VASc score (Figure 7a–c). Thus, we advised using the nomogram to decide whether and when to initiate reablation.

### 3.6. Risk Stratification and Its Separative Efficacy

**Figure 8:**
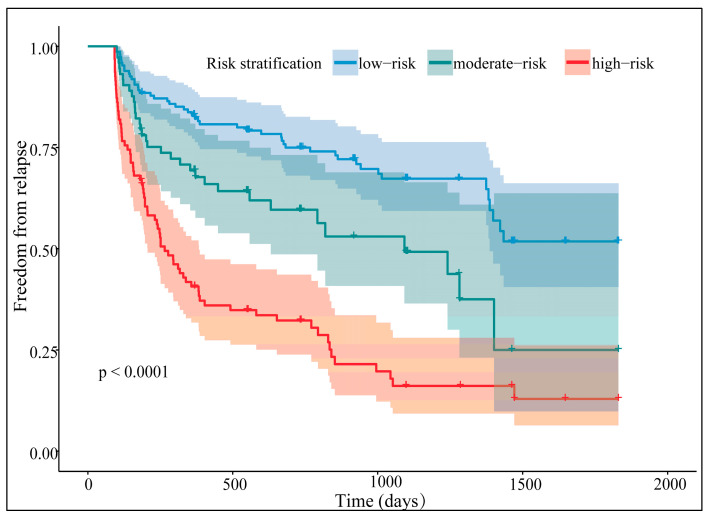
The nomogram-based risk stratification.

The *CatPredi* package identified two cutoffs for the total points in the training cohort: 103.3 and 173.4, separating patients into the low-, moderate-, and high-risk groups. After converging the two cohorts, the nomogram separated the outcomes distinctively (Figure 8, log-rank test: *p* < 0.0001). 

**Figure 9:**
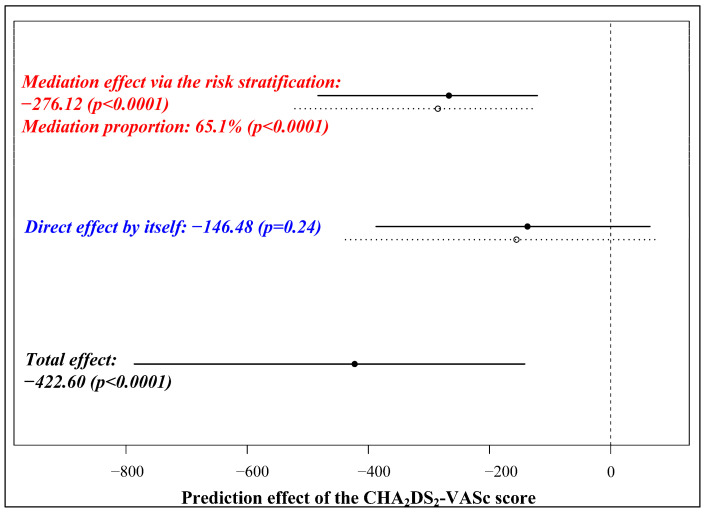
Mediation effect analysis of the CHA_2_DS_2_-VASc score.

The stakes of ablation failure were significantly higher in the moderate- and high-risk groups when compared with the low-risk group (HR for the moderate-risk group: 1.90, 95% CI (1.22–2.97), *p* = 0.005; HR for the high-risk group: 4.06, 95% CI (2.80–5.88), *p* < 0.0001). To compare the risk stratification methodologies, we also classified the CHA_2_DS_2_-VASc score, with one and two scores being the threshold. Two risk scores were linearly associated in terms of risk stratification (Mantel–Haenszel statistic: 26.04, *p* < 0.0001), while no agreement was achieved (weighted Kappa: 0.21, *p* < 0.0001). Moreover, the nomogram’s risk stratification mediated 65.1 percent of the CHA_2_DS_2_-VASc score’s prediction, with the latter posing no direct impact on the outcomes (Figure 9). Thus, the five-factor nomogram was a reliable prognostic tool to distinguish outcomes after PAF ablation.

## 4. Discussion

In the research, we first reported the development of a five-factor nomogram for predicting PAF ablation failure and its internal validation. The nomogram was moderately correlated with the CHA_2_DS_2_-VASc score but outperformed the latter significantly in discrimination and clinical utility. It also performed well in goodness-of-fit and calibration. We recommended setting 103.3 and 173.4 points as cutoffs for stratifying patients. The risk of frustrating ablation outcomes mounted hierarchically in line with the risk stratification. 

With the publication of various predictors for AF recurrence, the development of prediction models becomes an emerging scene. For instance, Zhao et al. reported a four-factor nomogram consisting of AF type, LA diameter, LVEF, and systemic inflammation score [25]. Based on NTproBNP, AF type, LAA volume, and LA volume, Zhou et al. built a deep learning-based model via a convolutional neural network [26]. Obviously, they shared the scheme to include AF type and the LA structural parameters while we focused on PAF patients and abandoned LA diameter. The contrast may be attributed to persistent AF having an overwhelmingly larger LA size than PAF. In our works, it made sense that the exclusion of persistent AF, which restricted the degree of LA dilatation, could at least partly account for the inability of LA diameter. Hypertension and obesity, whose effect on AF is highly mediated by LA dilatation, might be weakened simultaneously [11,27]. Additionally, the unidimensional measurement of LA size might overlook the virtual extent of enlargement, as it may be asymmetric, and the LA is not strictly spheric [28]. In summary, due to the study population and possible underestimation, LA dilatation was not a key predictor for AF recurrence in the research, let alone hypertension and BMI.

Despite the absence of LA size, the five-factor nomogram still provided evidence for LA remodeling, malfunction, and arrhythmogenesis, which interpreted its predictive effect. As its component, the prolongation of PWD is a noninvasive indicator of atrial conduction delay, rendering a substrate favoring atrial re-entry. It also unveils electro-anatomical remodeling, which facilitates AF initiation and perpetuation [29]. Unsurprisingly, it elevates the odds of poor ablation outcomes [30,31], as reaffirmed in the current study. Moreover, after adjusting for the other co-variates, PWDs in lead aVF and V1 were both eligible, implying that the electrical heterogeneity among PAF patients might be pivotal to prediction.

LAA, the reservoir of LA blood flow, is susceptible to a mild shift in LA status [32]. A decreased LAA emptying flow velocity reveals the LAA flow stasis, as well as its decompensation for a deteriorated LA pressure–volume relationship. As an indirect marker of LA dysfunction, it has surpassed LA diameter or some other structural parameters with limited evidence [33,34]. Likewise, in the current study, a flow velocity slower than 31 cm/s outperformed all the other candidates as the most contributive factor to the nomogram.

Renal insufficiency, with co-existing fluid overload, metabolic abnormalities, and activation of the renin-angiotensin-aldosterone system, may lift the incidence of atrial arrhythmias [35]. Its relation with LA dilatation and AF recurrence has been established in a large Asian population [19]. In addition, in PAF patients, a low eGFR may provide additional arrhythmogenesis other than pulmonary veins [20]. In our works, the eGFR decreased in tandem with LA enlargement (Figure 2), redemonstrating its contribution to structural remodeling. In addition, it fitted closer with PWD prolongation than LA diameter, which suggested a potential role in electrical remodeling and explained its eligibility.

Evidently, females suffer more AF recurrences than males after catheter ablation [18,36,37]. Some researchers attribute the phenomenon to females having higher LA volume and lower LA voltage, and hence more evidence for LA remodeling [37]. However, our works showed that the female sex had a smaller LA size and lower BMI than its counterpart (Figure 2), similar to that previously reported on a pooled population. Furthermore, it tended to have a narrower PWD. Though disfavoring electro-anatomical remodeling on the external signs, the female sex by itself was still an independent risk factor. Internal mechanisms, such as more ectopic activities, more frequent beating from pulmonary veins, and a higher burden of LA fibrotic remodeling, may help explain why females have lower ablation efficacy than males [38,39].

Regardless of the LA diameter, the five-factor nomogram provided a direct and indirect profile of LA, not to mention the perspectives besides LA, making itself a competitive prognostic tool. LA diameter might not be necessary for developing prediction models, especially among PAF patients.

As expected, the CHA_2_DS_2_-VASc score competed with the nomogram in prediction. The score contained several AF-related comorbidities and aging, setting the basis for prediction. Its concordance with LA remodeling further enhanced the predictive effect and explained the correlation between the two risk scores [40]. However, a high proportion of lone AF undermined its predictive power, as the incidence of comorbidities was low, and the left ventricular function was quite preserved. With no focus on comorbidities, the superiority of the nomogram might lie in the emphasis on the electrical heterogeneity among PAF patients and the indirect depiction of LA.

It is worth noting that the nomogram had space for promoting its performance, as we set the priority of application at a slight sacrifice of discrimination. In addition, it might harm patients when the practitioners set the threshold probabilities at high levels, indicating a relative shortage of specificity [41]. The P wave parameters we neglected may improve the nomogram’s power: P wave amplitude [42], PR interval [43,44], and inter-atrial block [30]. Despite the failure of LA diameter, LA structural parameters, including LA volume and its index, may still be crucial, as they provide an overall assessment of LA enlargement [45]. As a response to the excessive LA load, LAA enlargement is also a candidate. To make the decision wiser, we recommend developing more nomograms based on the predictors above. 

In the spectrum of AF development, PAF far precedes persistent AF in progressive LA remodeling. The latter manifests unique signs, such as less reliance on pulmonary veins, more ectopic foci, and more complicated LA substrates than the former [7,46]. Resultantly, the ablation strategies between the two vary a lot. Thus, excluding the AF type from model construction seems rational. Since we concentrated on PAF in the study, a prognostic tool targeting persistent AF is then required.

To ensure the sample size, we included patients receiving either cryoballoon or radiofrequency ablation. Virtually, the former guarantees a remarkable improvement over the latter, in terms of procedural time, lab efficiency, and even economic costs, with the overall efficacy and safety remaining similar [47]. Additionally, the generation of zero X-ray ablation is approaching, which highly attenuates radiological exposure to patients, thus decreasing the long-term incidence of malignancies [48]. Therefore, besides the model construction to optimize post-ablation decision-making, the evolving technology is believed to provide a brighter future for AF patients.

Though well-designed and conducted, the research had several limitations. First, we could not eliminate all the biases due to the retrospective nature. Second, all the conclusions were inferred from a relatively small-scaled population, which might cause data overfitting. Therefore, the nomogram was advised to be revalidated in a multicentered, prospective, and large-scale cohort. Third, though being limited by blinding, subjective bias in echo- and electro-cardiographic measurements was somewhat inevitable. Finally, the nomogram was not confident in covering persistent AF.

## 5. Conclusions

The study proposed a five-factor nomogram predictive of relapse after PAF ablation. The nomogram performed well among discrimination, goodness-of-fit, calibration, clinical utility, and separative efficacy. It would be helpful in clinical practice if the revalidation in a multicentered, prospective, and large-scale cohort was attained.

## Figures and Tables

**Table 1:** Demographic and clinical characteristics.

Characteristics	Relapse (*n* = 153)	Censoring (*n* = 162)	*p*
CHA_2_DS_2_-VASc score	2 (2)	1 (2)	<0.001
Age (years)	59.77 ± 11.93	58.35 ± 10.64	0.269
Sex (female), *n* (%)	75 (49.02)	46 (28.40)	<0.001
Hypertension, *n* (%)	85 (55.56)	75 (46.30)	0.100
Diabetes, *n* (%)	24 (15.69)	22 (13.58)	0.597
Stroke/TIA, *n* (%)	30 (19.61)	19 (11.73)	0.054
LVEDd (mm)	47.82 ± 4.10	48.06 ± 4.11	0.597
LVEF (%)	60.54 ± 5.74	61.33 ± 4.83	0.191
LA diameter (anterior-posterior) (mm)	36.59 ± 4.54	35.81 ± 4.38	0.123
LAA emptying flow velocity (cm/s)	39.58 ± 11.27	42.50 ± 11.74	0.025
eGFR (mL/(min·1.73 m^2^))	90.43 (27.99)	90.45 (18.29)	0.270
BMI (kg/m^2^)	23.95 ± 3.20	23.94 ± 2.72	0.973
NTproBNP, *n* (%)			0.076
~150 pg/mL	76 (49.67)	89 (54.94)	
150~ pg/mL	68 (44.45)	55 (33.95)	
missing data	9 (5.88)	18 (11.11)	
P wave duration			
Lead II (ms)	121.53 ± 18.32	118.65 ± 18.98	0.171
Lead III (ms)	111.99 ± 21.31	105.34 ± 21.13	0.006
Lead aVF (ms)	117.72 ± 19.19	113.75 ± 20.06	0.074
Lead V1 (ms)	106.52 ± 20.66	99.75 ± 19.05	0.003
Terminal negative phase in V1 (ms)	57 (21)	53 (21)	0.051
Medications			
ACEI/ARB, *n* (%)	47 (30.72)	45 (27.78)	0.566
beta-blockers, *n* (%)	31 (20.26)	32 (19.75)	0.910
amiodarone, *n* (%)	118 (77.12)	138 (85.19)	0.067
propafenone, *n* (%)	14 (9.15)	7 (4.32)	0.086
NOACs, *n* (%)	136 (88.89)	146 (90.12)	0.721
Ablation procedure			
radiofrequency, *n* (%)	61 (39.87)	69 (42.59)	0.624
cryoballoon, *n* (%)	92 (60.13)	93 (57.41)	0.624
procedural time (min)	144.95 ± 41.95	142.57 ± 43.33	0.620
Additional procedures, *n* (%)	23 (15.03)	26 (16.05)	0.804
superior vena cava isolation, *n* (%)	9 (5.88)	13 (8.03)	0.456
linear ablation, *n* (%)	5 (3.27)	2 (1.24)	0.271
cavo-tricuspid isthmus abaltion, *n* (%)	15 (9.80)	17 (10.49)	0.839

**Table 2:** Univariate, lasso-based, and stepwise Cox regression analyses.

	Univariate Analysis		Lasso-Cox	Stepwise Regression	
	HR (95% CI)	*p*	Shrunk HR	HR (95% CI)	*p*
Sex (female)	1.79 (1.23–2.62)	0.003	1.15	1.83 (1.23–2.71)	0.003
Hypertension (yes)	1.11 (0.76–1.63)	0.594			
eGFR < 65.8 mL/(min·1.73 m^2^)	2.89 (1.77–4.71)	<0.001	1.39	1.73 (1.01–2.96)	0.046
LA diameter > 33 mm	1.31 (0.83–2.09)	0.246			
LAA emptying flow velocity ≤ 31 cm/s	1.89 (1.22–2.94)	0.005	1.12	1.90 (1.22–2.98)	0.005
BMI > 26 kg/m^2^	1.37 (0.90–2.09)	0.143			
PWD in lead II ≥ 120 ms	1.86 (1.26–2.75)	0.002	1		
PWD in lead III ≥ 120 ms	1.66 (1.12–2.46)	0.011	1		
PWD in lead aVF ≥ 120 ms	2.21 (1.50–3.25)	<0.001	1.31	1.85 (1.23–2.79)	0.003
PWD in lead V1 ≥ 100 ms	2.12 (1.41–3.18)	<0.001	1.17	1.63 (1.06–2.51)	0.026
Terminal negative phase of PWD in lead V1 ≥ 60 ms	1.57 (1.07–2.30)	0.021	1		

**Table 3:** Overall evaluation of the five-factor nomogram and the CHA_2_DS_2_-VASc score.

Five-Factor Nomogram vs. CHA_2_DS_2_-VASc Score	IDI (95% CI)	*p*-Value	NRI (95% CI)	*p*-Value
1-year	0.108 (0.042–0.176)	<0.001	0.317 (0.120–0.423)	<0.001
2-year	0.137 (0.069–0.204)	<0.001	0.340 (0.188–0.443)	<0.001
3-year	0.156 (0.078–0.230)	0.002	0.366 (0.213–0.511)	0.002

**Table 4:** The AIC, BIC of predictors, the five-factor nomogram, and the CHA_2_DS_2_-VASc score.

Models	AIC	BIC
Five-factor nomogram	1013.40	1030.37
Sex	1041.50	1044.90
LAA emptying flow velocity	1043.34	1046.74
eGFR	1035.97	1039.37
PWD in lead aVF	1034.46	1037.86
PWD in lead V1	1036.54	1039.94
CHA_2_DS_2_-VASc score	1042.86	1046.25

## Data Availability

The data presented in the study are available on reasonable request from the corresponding author. The data are not publicly available due to respect for patients’ privacy.
